# Cost-effectiveness of a screening strategy for Q fever among pregnant women in risk areas: a clustered randomized controlled trial

**DOI:** 10.1186/1472-6874-10-32

**Published:** 2010-11-01

**Authors:** Janna M Munster, Alexander CAP Leenders, Wim van der Hoek, Peter M Schneeberger, Ariene Rietveld, Josien Riphagen-Dalhuisen, Ronald P Stolk, Carl JCM Hamilton, Esther de Vries, Jamie Meekelenkamp, Jerome R Lo-Ten-Foe, Albertus Timmer, Lolkje TW De Jong - van den Berg, Jan G Aarnoudse, Eelko Hak

**Affiliations:** 1 University of Groningen, University Centre for Pharmacy, PharmacoEpidemiology & PharmacoEconomics, Antonius Deusinglaan 1, 9713 AV, Groningen, The Netherlands; 2 University Medical Centre Groningen, University of Groningen, Department of Epidemiology, Hanzeplein 1, 9700 RB, Groningen, The Netherlands; 3 University Medical Centre Groningen, University of Groningen, Department of Obstetrics and Gynaecology, Hanzeplein 1, 9700 RB, Groningen, The Netherlands; 4 Jeroen Bosch Hospital, Department of Medical Microbiology and Infection Prevention, Tolbrugstraat 11, 5211 RW, 's-Hertogenbosch, The Netherlands; 5 National Institute for Public Health and the Environment, Antonie van Leeuwenhoeklaan 9, 3721 MA, Bilthoven, The Netherlands; 6 Public Health Department ''Hart voor Brabant'', Vogelstraat 2, 5212 VL 's-Hertogenbosch, The Netherlands; 7 Jeroen Bosch Hospital, Department of Obstetrics and Gynaecology, Tolbrugstraat 11, 5211 RW, 's-Hertogenbosch, The Netherlands; 8 Jeroen Bosch Hospital, Department of Paediatrics, Tolbrugstraat 11, 5211 RW, 's-Hertogenbosch, The Netherlands; 9 University Medical Centre Groningen, University of Groningen, Department of Medical Microbiology, Hanzeplein 1, 9700 RB, Groningen, The Netherlands; 10 University Medical Centre Groningen, University of Groningen, Department of Pathology and Medical Biology, Hanzeplein 1, 9700 RB, Groningen, The Netherlands

## Abstract

**Background:**

In The Netherlands the largest human Q fever outbreak ever reported in the literature is currently ongoing with more than 2300 notified cases in 2009. Pregnant women are particularly at risk as Q fever during pregnancy may cause maternal and obstetric complications. Since the majority of infected pregnant women are asymptomatic, a screening strategy might be of great value to reduce Q fever related complications. We designed a trial to assess the (cost-)effectiveness of a screening program for Q fever in pregnant women living in risks areas in The Netherlands.

**Methods/design:**

We will conduct a clustered randomized controlled trial in which primary care midwife centres in Q fever risk areas are randomized to recruit pregnant women for either the control group or the intervention group. In both groups a blood sample is taken around 20 weeks postmenstrual age. In the intervention group, this sample is immediately analyzed by indirect immunofluorescence assay for detection of IgG and IgM antibodies using a sensitive cut-off level of 1:32. In case of an active Q fever infection, antibiotic treatment is recommended and serological follow up is performed. In the control group, serum is frozen for analysis after delivery. The primary endpoint is a maternal (chronic Q fever or reactivation) or obstetric complication (low birth weight, preterm delivery or fetal death) in Q fever positive women. Secondary aims pertain to the course of infection in pregnant women, diagnostic accuracy of laboratory tests used for screening, histo-pathological abnormalities of the placenta of Q fever positive women, side effects of therapy, and costs. The analysis will be according to the intention-to-screen principle, and cost-effectiveness analysis will be performed by comparing the direct and indirect costs between the intervention and control group.

**Discussion:**

With this study we aim to provide insight into the balance of risks of undetected and detected Q fever during pregnancy.

**Trial registration:**

ClinicalTrials.gov, protocol record NL30340.042.09.

## Background

Q fever, a zoonosis caused by *Coxiella burnetii *(*C. burnetii*), primarily infects ruminants and rodents [1]. Especially pregnancy products of infected animals like placentas and amniotic fluid can contain high numbers of bacteria. After drying, the organism spreads in aerosols and remains virulent for months. Humans are infected by inhalation of these contaminated aerosols. Most of the infected patients are either asymptomatic or present with a mild flu-like illness. However, Q fever may pose a serious threat to certain groups at risk, including pregnant women, immune compromised hosts and individuals with pre-existing cardiac valve or vascular defects [1,2]. In The Netherlands, the number of human cases of Q fever has dramatically increased from around 12 cases each year before 2007 to more than 2300 cases in 2009 [3-5]. This observation has led to several meetings of the Dutch Outbreak Management Team (OMT) of the Ministry of Health to curb the epidemic. Studies revealed that the epidemic among Dutch inhabitants was a result of Q fever outbreaks on dairy goat farms [6].

Pregnant women are by far the largest risk group in size. When infected by *C. burnetii*, most pregnant women will remain asymptomatic: percentages up to 90% have been described compared to 60% in the general population [7,8]. Notably, serious complications due to Q fever seem to occur more frequently during pregnancy if the infection is undetected and untreated. Pregnant women have an increased risk to develop chronic Q fever or to reactivate a past infection [9,10]. Furthermore, obstetric complications related to *C. burnetii *infection have been described. A landmark study from France showed obstetric complications including spontaneous abortion, preterm delivery, intrauterine growth restriction, oligohydramnios and fetal death in 81% of the 53 women who were positive for Q fever and not sufficiently treated with antibiotics [10]. However, because of the retrospective design selection bias might have led to overestimation of the complication prevalence. In a Canadian cohort study in an affected area, 3.8% of parturient women had evidence of previous exposure to *C. burnetii*. These women had higher risks for adverse obstetrical outcome in terms of premature delivery and prior or current neonatal death [11]. Little is known about the chances of vertical transmission from mother to child. Transmission across the placenta, transmission by inhalation of infected amniotic fluid or by ingestion of infected milk cannot be excluded.

Because most infected pregnant women remain asymptomatic, one of the suggested measures to prevent obstetric complications and maternal chronicity concerns a screening strategy. However, because of lack of information on the prevalence during pregnancy and lack of randomized controlled trials weighing potential benefits and risks associated with screening, evidence for its potential impact is scarce. The Health Council of The Netherlands therefore advised the Ministry of Health in 2008 to facilitate studies to inform decision makers. We therefore designed a trial to assess the effects of a screening policy for Q fever in pregnant women from areas with large numbers of Q fever cases on the pregnancy outcome and cost-effectiveness from a societal and health care perspective. The study will primarily provide insights into the balance of risks of undetected and detected Q fever during pregnancy.

## Methods/Design

**Figure 1:**
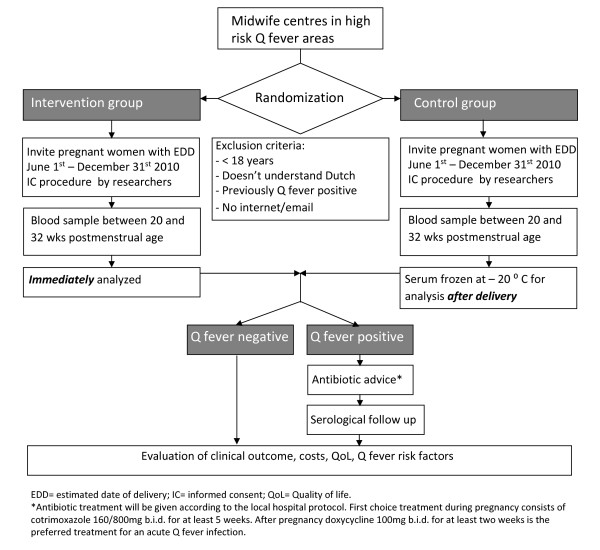
**Timing and phasing of the study**.

Since ethical issues surrounding randomization of the individual pregnant woman for a Q fever screening or non-screening strategy could seriously threaten approval by an ethics committee, we designed a clustered randomized controlled trial in which primary care midwife centres are randomized to recruit either pregnant women for the control group or for the intervention group. In this way, the choice for either strategy by individual eligible women was avoided. Timing and phasing after eligibility checks are shown in figure 1. The study will be conducted according to the principles of the Declaration of Helsinki. The study protocol is approved by the Medical Ethical Review Board of the University Medical Centre Groningen. The study protocol is registered at http://ClinicalTrials.gov, protocol record NL30340.042.09. The inclusion of participants started in April 2010.

The conduct of the trial is currently supported by the Royal Dutch Society for Midwifery (KNOV), the professional organization of midwives. Midwife centres in risk areas for Q fever (incidence in 2009 of more than 50:100,000 inhabitants according to the National Institute for Public Health and the Environment (RIVM)), were primarily invited to facilitate inclusion of participants. During spring 2010 we expanded the area based on the incidence of 2010. All obstetricians, paediatricians, medical microbiologists and pathologists in these areas were informed about the study.

### Inclusion criteria

Pregnant women, 18 years of age or older, with an estimated date of delivery between June 1^st ^and December 31^st ^2010, and under supervision of a midwife in primary health care are eligible for inclusion. In The Netherlands, midwives working in primary health care are allowed to only supervise uncomplicated, singleton pregnancies. It is estimated that approximately 10,000 eligible pregnant women live in the Q fever affected areas.

### Exclusion criteria

Women who do not have access to internet and/or an email address are excluded because data collection is web-based. In addition, women who are unable to understand Dutch or to give informed consent, or who have previously been tested positive for Q fever are ineligible for participation into the study.

### Experimental procedure

#### Intervention group

**Figure 2:**
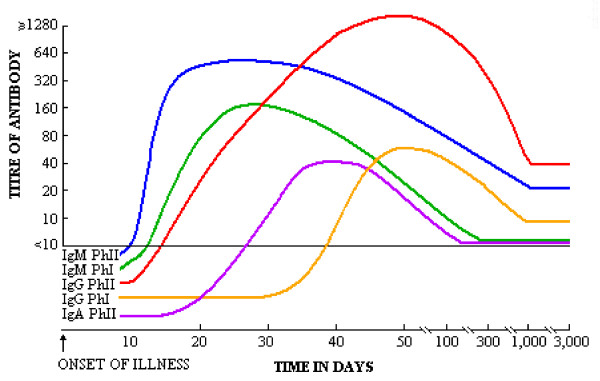
**Idealised antibody responses in acute Q fever as measured by IFA^13^**.

Participants who are recruited by a midwife centre randomized for the intervention group are asked for a blood sample around 20 weeks postmenstrual age. If possible the visit is combined with the routine structural ultrasound scan around that time. If the participant is included after 20 weeks, the blood sample will be taken as soon as possible after inclusion. The sample will immediately be tested for antibodies against *C. burnetii *in the laboratory of the Jeroen Bosch Hospital which has analyzed most samples during the epidemic in 2007, 2008, and 2009. Serologic diagnosis of Q fever will be made by indirect immunofluorescence assay (IFA), the reference method for serodiagnosis of Q fever [12]. Both IgM and IgG antibodies against phase I and phase II antigens are measured according to the manufacturer's instructions (Focus Diagnostics, Cypress, CA, USA). Titres ≥ 1:32 are considered positive. All positive samples will be fully titrated to reduce the chance of treatment in false positives. In general, the first antibody to appear in acute Q fever patients is IgM phase II, followed by a more or less simultaneous IgG phase II and IgM phase I response and subsequent appearance of IgG phase I antibodies (see figure 2) [13]. This time-dependent serologic profile allows us to discriminate between a recent acute infection, a past infection, and a chronic infection. If the pregnant woman does not have evidence for an acute, past or chronic Q fever infection, standard care will be provided.

In case of acute Q fever, the participant will be referred to an obstetrician, and treatment will be advised according to the local hospital protocol. In the literature, the first choice treatment of Q fever during pregnancy is oral cotrimoxazole (sulfamethoxazole/trimethoprim) for at least 5 weeks [10]. Antibiotic treatment, further obstetrical care and serological follow up will be supervised by the obstetrician in collaboration with the medical microbiologist. The current routine for pregnant women being treated for acute Q fever is to perform monthly blood analyses to detect the development of chronic Q fever. If the titres decline, the frequency of these controls is scaled down to once every two months during pregnancy, and at 3, 6 and 12 months after delivery. If chronic Q fever develops, treatment will be continued until the end of pregnancy followed by bactericidal treatment with doxycycline and hydroxychloroquine after delivery. In Q fever cases placentas will be collected for polymerase chain reaction (PCR) and histo-pathology.

If there is evidence for a past infection, no treatment is started. However, midwives will be advised to perform an extra serological analysis later in pregnancy to exclude reactivation.

#### Control group

Participants who are recruited by a midwife centre randomly allocated to the control group will also be asked for a blood sample around 20 weeks postmenstrual age. These blood samples will be stored at -20°C, and analyzed for *C. burnetii *after delivery. In case of a positive test, the participant's general practitioner will be advised to perform an extra serological analysis to exclude chronic Q fever. Antibiotic treatment will be started if needed according to the local protocol.

### Neonates

All neonates born to Q fever positive mothers will receive care according to the local hospital protocols. The Section for Paediatric Infectious Diseases and Immunology of the Dutch Paediatric Society has formulated a consensus guideline for neonates born to Q fever positive women during pregnancy [14]. The guideline advices PCR at birth and one month of age, and serological follow up until 18 months of age in case of active maternal Q fever during pregnancy to diagnose and treat potential mother-to-child transmission. Preventive antibiotic treatment is not advised. Breastfeeding is contraindicated if maternal serum or milk is *C. burnetii *PCR positive. Breastfeeding might also be contraindicated in case of maternal medication use.

### Randomization procedure

Participating midwife centres are randomized to include either pregnant women for the control group or for the intervention group. Randomization is stratified according to the risk of contracting a *C. burnetii *infection as determined by the number of goat farms in the neighbourhood (registration by Statistics Netherlands (CBS)), and by the size of the midwife centre.

### Inclusion of participants

Pregnant women are invited by their midwife to participate in the study. The informed consent procedure will be performed by the researchers.

### Data collection

Data will be collected in four ways using a structured case record file:

**1. Serological samples **will be collected at the time points described in the section Experimental procedure, and will be analyzed in the laboratory of the Jeroen Bosch Hospital.

**2. Questionnaires**; two questionnaires will be filled out by the participant and one will be filled out by the midwife/obstetrician.

At baseline, when the participant is included in the trial, a questionnaire is completed by all participants including questions about the current pregnancy, outcome of previous pregnancies, smoking and alcohol habits, co morbidities, medication use and demographic characteristics. With this questionnaire risk factors are assessed for complicated pregnancy outcome. After delivery all relevant outcome data on obstetric complications are collected by a questionnaire completed by the midwife. Questionnaires for participants who are referred to a hospital during pregnancy or delivery, are filled out by the obstetrician. During follow up, all health care and potential cost data will be measured by a third questionnaire completed by the participant one month after delivery. With this questionnaire we will also verify symptoms during pregnancy, health-related quality-of-life (using EQ5D [15]), depressive symptoms and fatigue (using the Shortened Fatigue Questionnaire [16]), potential long-term consequences of Q fever, tolerance to antibiotic treatment and problems and development of the newborn. Furthermore, the risk for Q fever infection will be assessed.

**3. PCR and histo-pathology of the placenta **will be performed by the local microbiologists and pathologists. Re-evaluation of the histological slides will be performed by one pathologist at the University Medical Centre Groningen.

**4. Medical data in primary care; **data on the health status of the participant or the newborn is collected from medical files of the general practitioner.

### Outcome measures

The primary endpoint is a maternal (chronic Q fever or reactivation) or obstetric complication (low birth weight, preterm delivery or fetal death) after the first trimester of pregnancy in Q fever positive women.

The secondary endpoints are direct and indirect costs of the screening program compared to costs of complications which could be prevented by screening. Furthermore, we aim to assess the course of infection in pregnant women, the accuracy of the diagnostic tests used for screening, histo-pathological abnormalities of the placenta of Q fever infected women, and side effects associated with treatment.

### Withdrawal of individual participants

Participants are informed that they can withdraw from the study at any time point, without giving a reason for withdrawal. If the blood sample has already been taken, participants will be asked to give permission for collecting data on obstetric outcome. Participants who withdraw will receive regular health care according to the local protocols.

### Sample size calculation and statistics

Based on the literature and pilot data from The Netherlands, we expect that 12% of pregnant women in the high-risk areas will have serological evidence for a Q fever infection [17,18]. Of these women, we conservatively estimate that 25% will develop complications, so 3% of women will have the primary outcome. Assuming a reduction of the complication rate of 50% by early detection with screening during pregnancy, we will need a participation of at least 3,400 participants with complete follow up (statistical power of 80 percent, p ≤ 0.05). Assuming a loss to follow up of 10% and to allow for a small clustering effect, we aim to include 4,000 participants.

Data will be analyzed according to intention-to-screen principles. A two-sided p-value of 0.05 or less will be considered to indicate statistically significant. Descriptive statistics concerning the distributions of the predictor variables and outcome variables will be performed using the software SPSS for windows (version 16). For univariate analysis the chi-square test and Fischer's exact test will be used to compare proportions. For variables with a normal distribution, differences will be analysed with Student's t-test. In case of non-parametric distribution, differences between populations will either be evaluated using the Mann-Whitney-U test or the data will be log-transformed to obtain a normal distribution. Relative risks as well as absolute risk reductions and numbers needed to treat will be estimated with their corresponding 95% confidence intervals. Possible clustering of outcome data will be taken into account using generalised estimating equations (GEE) modelling.

### Economic evaluation

The study will primarily provide insights into the economical balance of undetected and detected Q fever during pregnancy. The economic evaluation will be performed from a societal and health care perspective. Direct medical and non-medical costs (laboratory costs, costs of health care following positive screening, time, and travel costs) as well as indirect costs (loss of productivity) will be taken into account. The time horizon will be from taking the blood sample until one month after delivery for measured and calculated costs and until one year after delivery for estimated costs. Data on health care use and productivity loss will be collected by questionnaires. Unit costs will be based on the Dutch 2004 guidelines for costing in health care research and indexed for base year 2010 using yearly general consumer price indices [19].

## Discussion

Right at the beginning of the first Q fever outbreak in The Netherlands in 2007, the government and health care providers assessed the risk of Q fever related to the outcome of pregnancies [20]. In all, only research from France is currently available on the risks of Q fever during pregnancy and the benefits of long-term antibiotic treatment [10]. There is, however, a lack of data on the prevalence and the risk of Q fever infection and the impact of antibiotic treatment during pregnancy in other countries such as The Netherlands. Therefore, in December 2008 the Dutch Health Council advised the Ministry of Health not to screen for Q fever during pregnancy until additional scientific data would be available to support screening [20]. The Dutch outbreak is an opportunity to gain more knowledge in this field. Therefore we will conduct the study described previously, to provide insights into the balance of risks of undetected and detected Q fever during pregnancy. By the end of February 2011 all data will be available for analysis. First results are expected in spring 2011.

## Competing interests

The authors declare that they have no competing interests.

## Authors' contributions

JMM participated in the design of the study, drafted the manuscript and will perform the study; ACAPL participated in the design of the study as an expert on laboratory testing and will perform the diagnostic analysis and therapeutical advices; WvdH participated in the design of the study as an expert on national public health care and will perform data on Q fever risk areas; PMS participated in the design of the study as an expert on laboratory testing and will perform the diagnostic analysis and therapeutical advices; AR participated in the design of the study as an expert on local health care in the most affected areas of The Netherlands; JRD participated in the design of the study for approval from the ethical board; RPS participated in the design of the study and performed the part about statistical analysis; CJCMH participated in the design of the study as an expert on obstetric care; EdV participated in the design of the study as an expert on paediatrics; JM will be responsible for the logistics surrounding laboratory testing; JRLTF participated in the design of the study as an expert on laboratory testing; AT participated in the design of the study as an expert on histo-pathology and will perform the re-evaluation of the histo-pathological examination of the placentas; LTWdJvdB participated in the design of the study as an expert on pharmacology during pregnancy; JGA participated in the design of the study as an expert on obstetric care and will supervise the analysis and report; EH initiated and designed the study, and will supervise the data collection, analysis and report. All authors revised the draft manuscript and approved the final manuscript.

## Pre-publication history

The pre-publication history for this paper can be accessed here:

http://www.biomedcentral.com/1472-6874/10/32/prepub
